# Imbalanced plasma ACE and ACE2 level in the uremic patients with cardiovascular diseases and its change during a single hemodialysis session

**DOI:** 10.1080/0886022X.2017.1398665

**Published:** 2017-11-20

**Authors:** Chung-Wei Yang, Li-Che Lu, Chia-Chu Chang, Ching-Chang Cho, Wen-Yeh Hsieh, Chin-Hung Tsai, Yi-Chang Lin, Chih-Sheng Lin

**Affiliations:** a Department of Biological Science and Technology, National Chiao Tung University, Hsinchu, Taiwan;; b Division of Nephrology, Department of Internal Medicine, National Taiwan University Hospital Hsinchu Branch, Hsinchu, Taiwan;; c Division of Nephrology, Department of Internal Medicine, Shin Kong Wu Ho-Su Memorial Hospital, Taipei, Taiwan;; d Division of Nephrology, Department of Internal Medicine, Changhua Christian Hospital, Changhua, Taiwan;; e School of Medicine, Chung-Shan Medical University, Taichung, Taiwan;; f Division of Chest Medicine, Department of Internal Medicine, Hsinchu Mackay Memorial Hospital, Hsinchu, Taiwan;; g Department of Senior Citizen Service Management, Minghsin University of Science and Technology, Hsinchu, Taiwan

**Keywords:** Angiotensin converting enzyme, angiotensin converting enzyme II, hemodialysis, renin-angiotensin system, uremic patient, cardiovascular diseases

## Abstract

**Background:** The renin-angiotensin system (RAS) has significant influences on heart and renal disease progression. Angiotensin converting enzyme (ACE) and angiotensin converting enzyme II (ACE2) are major peptidases of RAS components and play counteracting functions through angiotensin II (Ang II)/ATIR and angiotensin-(1–7) (Ang-(1–7))/Mas axis, respectively.

**Methods:** There were 360 uremic patients on regular hemodialysis (HD) treatment (inclusive of 119 HD patients with cardiovascular diseases (CVD) and 241 HD patients without CVD and 50 healthy subjects were enrolled in this study. Plasma ACE, ACE2, Ang II and Ang-(1–7) levels of the HD patients were determined.

**Results:** We compared pre-HD levels of plasma ACE, ACE2, Ang II and Ang-(1–7) in the HD patients with and without CVD to those of the controls. The HD patients, particularly those with CVD, showed a significant increase in the levels of ACE and Ang II, whereas ACE2 and Ang-(1–7) levels were lower than those in the healthy controls. Therefore, imbalanced ACE/ACE2 was observed in the HD patients with CVD. In the course of a single HD session, the plasma ACE, ACE/ACE2 and Ang II levels in the HD patients with CVD were increased from pre-HD to post-HD. On the contrary, ACE2 levels were decreased after the HD session. These changes were not detected in the HD patients without CVD.

**Conclusions:** Pathogenically imbalanced circulating ACE/ACE2 was detected in the HD patients, particularly those with CVD. HD session could increase ACE/Ang II/AT1R axis and decrease ACE2/Ang-(1–7)/Mas axis activity in the circulation of HD patients with CVD.

## Introduction

The renin-angiotensin system (RAS) has a significant influence on renal disease progression [1]. In the RAS, two of the most important and commonly studied axes are triggered by two peptidases, angiotensin-converting enzyme (ACE) and angiotensin-converting enzyme II (ACE2). ACE plays physiological functions through angiotensin II (Ang II) and angiotensin type I receptor (ATIR), named classical ACE/Ang II/ATR1 axis. ACE2 acts the opposite functions to ACE/Ang II/ATR1 axis through angiotensin-(1–7) (Ang-(1–7)) and its receptor Mas, named non-classical Ang-(1–7)/Mas axis [2,3]. ACE/Ang II/AT1R axis promotes sodium and water retention, vasoconstriction, inflammation and fibrosis. ACE2, the other crucial peptidase in RAS, was discovered in 2000 [4,5], which indicated that the RAS is more complex than was previously imagined. The main functions of ACE2 in RAS are the synthesis of inactive angiotensin-(1–9) (Ang-(1–9)) from angiotensin I (Ang I) and the catabolism of Ang II to form Ang-(1–7). Specifically, ACE2/Ang-(1–7)/Mas axis induces natriuresis, vasodilation, anti-inflammation and anti-fibrosis pathogenesis. These processes contribute to protecting the kidneys from damage [3,6–8]. Furthermore, accumulating evidence indicate that imbalanced ACE and ACE2 (ACE/ACE2) regulates the production and accumulation of Ang II and that ACE2 deficiency leads to higher Ang II concentrations [9–14]. Based on crucial roles of RAS in the pathogenic development of chronic kidney disease (CKD), we tested the hypothesis whether plasma ACE and ACE2 levels might be significantly changed in the patients with end-stage CKD (ESRK), i.e., uremic patients.

Cardiovascular diseases (CVD) are the most common comorbidity and cause of mortality in HD population [15,16]. Therefore, we aimed to evaluate the associations of ACE and ACE2 change in the HD patients with CVD. We determined and compared plasma levels of ACE, ACE2, Ang II and Ang-(1–7) between the HD patients with and without CVD and healthy controls in this study. In addition, the changes of circulating ACE/ACE2 ratio were analyzed in relation to CVD in the HD patients during a signal HD session.

## Materials and methods

### Patient populations

In this prospective study, we enrolled 360 HD patients who received the hemodialysis in a single hemodialysis unit of a medical center of the Shin Kong Wu Ho-Su Memorial Hospital, Taipei, Taiwan between January 2014 and December 2014 [17]. All of the patients are Taiwanese with CKD stage 5 (GFR < 10 mL/min) and they were consecutively patients in the hemodialysis unit. Mean dialysis vintage of the 360 HD patients was 7.7 years. The primary diseases of the HD population include diabetes mellitus, chronic pyelonephritis, polycystic renal disease, glomerulonephritis and hypertensive nephropathy. They were divided into two groups: (1) HD patients with CVD group (clinical symptoms and/or previous history of CVDs include coronary artery diseases with/without myocardial infarction, congestive heart failure, prior cerebrovascular accident/stroke and hypertension) and (2) HD patients without CVD group (include diabetes mellitus, chronic pyelonephritis, polycystic renal disease, glomerulonephritis, etc.). Among of 360 patients, 119 (33.1%) were in CVD group and the other 241 (66.9%) patients were in without CVD group. The treatment of angiotensin II receptor blockers (ARBs) and/or calcium channel blockers (CCBs) was required for hypertension control in the HD patients. The medications in the HD patients also included recombinant human erythropoietin and/or statins at standard doses. We excluded the HD patients (1) with age <20 years, (2) HD duration <0.25 years, (3) ACE inhibitor (ACEI) uses, (4) positive serum marker of Human Immunodeficiency Virus (HIV) and (5) alcohol or illicit drug abusers. In this study, the comparison between the HD patients with and without CVD groups is a *post-hoc* analysis.

Fifty healthy subjects (aged 64.0 ± 13.2; range, 31–87 years, 23 women) who received physical examination alone at Shin Kong Wu Ho-Su Memorial Hospital, nonsmoking volunteers matched for age and gender with HD patients were served as controls. The healthy subjects were also carefully screened for any symptoms and signs indicative of either renal or CVD, neither of which were found on both clinical examination and standard laboratory tests. Additionally, all of them were on a regular diet, were receiving no drugs or vitamin supplements at the time of the study.

The study protocol (IRB20130811R) was reviewed and approved by the Bioethics Committee of Shin Kong Wu Ho-Su Memorial Hospital (Taipei, Taiwan) and written informed consent was obtained from all participants.

### HD procedure

The HD patients were routinely dialyzed three times a week for 3–4 h with a polysulfone hollow-fiber membrane of Fresenius Medical Care 4008 S (Deutschland GmbH, Bad Homburg, Germany), bicarbonate dialysate and standard heparin anticoagulation. Reverse osmosis was used for water treatment and the dialysate was regularly checked for the presence of endotoxin. Dialysis adequacy was evaluated by measuring Kt/V (*K* – dialyzer clearance of urea; *t* – dialysis time; *V* – volume of distribution of urea, approximately equal to patient’s total body water). Dialysis aimed at a urea reduction ratio 0.65 and the value of Kt/V of 1.2. Patients had fluid removal down to their dry weight. Heparinized, fasting venous blood was sampled just before and at the end of the HD session (as pre-HD and post-HD, respectively). The HD patients were investigated on a mid-week day, before a dialysis session. Venous blood samples were collected from HD patients and from controls in the morning after an overnight fast and medicine. The blood samples were centrifuged at 1000*g* for 10 min and plasma fractions were immediately stored at −80 °C until used for biochemical measurements.

### Hematological and biochemical assays

Hematological and biochemical parameters, including total protein, albumin, fasting blood glucose (glucose AC), total cholesterol, triglyceride, alanine aminotransferase (ALT), aspartate aminotransferase (AST), mean corpuscular volume (MCV), hematocrit (Hct), platelet, hemoglobin (Hb), blood urea nitrogen (BUN), uric acid, creatinine, sodium (Na), potassium (K), calcium (Ca), phosphorus (P) and the number of red blood count (RBC) as well as white blood count (WBC), were determined by routine procedures using an automated analyzer (ADVIA1800; SIEMENS, Munich, Germany).

### Plasma determinations of ACE and ACE2

Plasma ACE and ACE2 activities were assayed using the fluorogenic substrates Mca-YVADAPK and Mca-APK-Dnp (AnaSpec, San Jose, CA), respectively, according to our previous report [18] and report by Anguiano et al. [19] with slight modifications. The assay was performed in a microquartz cuvette with 20 μL of plasma and 2 μL of the fluorogenic substrates (stock concentration: 4 mM ACE substrate/1.5 mM ACE2 substrate) in ACE or ACE2 assay buffer (a total of reaction volume is 300 μL). The reaction was detected every 45 s with a time interval of 15 s during 1 h using a fluorescence reader at 330/390 nm. Each sample was detected in duplicate and normalized with a postive control in the same plate. All samples were fitted and plotted using Grafit version 4.0 (Sigma-Aldrich, St. Louis, MO), and enzyme activity was expressed as RFU/μL/h. The samples were also incubated with the above-mentioned reaction mixture in the presence of 1 μM captopril (Sigma-Aldrich; a specific ACE inhibitor) or 1 μM DX600 (AnaSpec; a specific ACE2 inhibitor) for ACE and ACE2 activity assay, respectively.

### Plasma measurements of Ang II and Ang-(1–7)

The concentration of Ang II and Ang-(1–7) in the plasma was determined using a human Ang II ELISA kit (MBS703599; MyBioSource, San Diego, CA) and Ang-(1–7) ELISA kit (MBS720194; MyBioSource, San Diego, CA), respectively. Each of the samples was determined by the same volume in duplicate. The measured protocols were performed according to the manufacturer’s instructions. Briefly, unlabeled mouse Ang II or Ang-(1–7) in the samples and biotin-labeled mouse Ang II or Ang-(1–7) were added to anti-mouse Ang II or Ang-(1–7) antibody coated on ELISA plates. After washing, samples were incubated with avidin conjugated to horseradish peroxidase (HRP), followed by addition of the substrate solution, tetramethylbenzidine (TMB), to develop the signal absorbance, which was measured at 450 nm. The intensity of color developed was inversely proportional to the concentration of Ang II or Ang-(1–7) in the samples. The results of serum sample assays were expressed in ng of per mL of plasma.

### Statistical analysis

Statistical analyses were performed using the SPSS statistical software package version 19.0 for Windows (SPSS, Chicago, IL). All parameters are presented as the mean with the standard deviation (SD). Clinical features were compared between the groups using the unpaired Student’s *t* test for the data with normally distributed continuous variables. Correlations were determined using Pearson’s correlation matrix. A value of two-tailed *p* < .05 was considered statistically significant.

The association between each variable and hot flash status was determined by multivariate linear regression analysis. The percentage difference in each variable was calculated using the formula (100*(exp(β) − 1)) and 95% CI for interpreting coefficients in the multivariate linear regression model. Sample size is ≥259 patients performed with the G*Power version 3.1.9.2 (Universitat Kiel, Kiel, Germany) software, *α* = 0.05, power of 0.9, and test and sample loss of 20%.

## Results

### General clinical characteristics of the uremic (HD) patients

The clinical characteristics of study subjects, including 360 HD patients and 50 healthy controls are listed in Table 1. Generally, glucose AC and uric acid in the HD patients were significantly higher than those in the controls (*p <* .001), whereas a lower Hct, platelet, Hb and the number of RBC was detected in the HD patients compared with those in the control (*p <* .001). The levels of total protein, albumin, total cholesterol, triglyceride, MCV and the number of WBC in both groups were insignificantly different (Table 1). Two major uremic toxins, BUN (71.1 ± 17.5 vs. 13.9 ± 5.0 mg/dL, *p <* .001) and creatinine (9.08 ± 2.10 vs. 0.87 ± 0.33 mg/dL, *p <* .001) in the HD patients were markedly higher than those in the healthy controls.

**Table 1.** Clinical and biochemical characteristics and risk factors of the HD patients and controls.

	Controls	HD patients	*p* Value
Number	50	360	
Gender (M/F)	27/23	181/179	
Age (years)	63.6 ± 13.9	66.0 ± 13.5	.246
Weight (kg)	57.3 ± 13.0	61.6 ± 39.7	.442
Total protein (g/dL)	6.88 ± 0.43	6.92 ± 0.52	.632
Albumin (g/dL)	4.06 ± 0.33	4.05 ± 0.32	.760
Glucose AC (mg/dL)	97.4 ± 27.4	126 ± 59***	<.001
Total Cholesterol (mg/dL)	168.3 ± 31.8	161 ± 38	.172
Triglyceride (mg/dL)	137.2 ± 77.3	149 ± 97	.405
AST (U/L)	19.0 ± 6.9	19.7 ± 8.2	.542
ALT (U/L)	16.1 ± 8.1	17.5 ± 10.1	.356
Hct (%)	39.3 ± 3.8	31.7 ± 3.1***	<.001
MCV (fL)	91.3 ± 6.5	92.5 ± 7.1	.228
Platelet (×10^3^/μL)	241 ± 78.1	199 ± 62***	<.001
Hb (g/dL)	12.1 ± 1.3	10.4 ± 1.1***	<.001
WBC (×10^3^/μL)	6.8 ± 2.5	7.2 ± 2.3	.251
RBC (×10^6^/μL)	4.3 ± 0.5	3.45 ± 0.44***	<.001
Uric acid (mg/dL)	4.55 ± 0.82	6.95 ± 1.26***	<.001
BUN (mg/dL)	13.9 ± 5.0	71.1 ± 17.5***	<.001
Creatinine (mg/dL)	0.87 ± 0.33	9.08 ± 2.10***	<.001
Na (meq/L)	–	137 ± 3	–
K (meq/L)	–	4.68 ± 0.66	–
Ca (mg/dL)	–	7.2 ± 2.41	–
P (mg/dL)	–	6.92 ± 0.52	–
HD treatment (years)	–	7.71 ± 6.59	–
Kt/V	–	1.63 ± 0.24	–

Data were expressed as mean ± SD. Biochemical data were detected before an HD session.

*** *p* < .001, Controls vs. HD patients, unpaired Student’s t test.

ALT: alanine aminotransferase; AST: aspartate aminotransferase; BUN: blood urea nitrogen; Glucose AC: fasting blood glucose; Hb: hemoglobin, Hct: hematocrit; HD: hemodialysis; Kt/V: (*K* – dialyzer clearance of urea; *t* – dialysis time; *V* – volume of distribution of urea, approximately equal to patient’s total body water); MCV: mean corpuscular volume; RBC: red blood count; WBC: white blood count.

The clinical characteristics of HD patients with CVD (*n* = 119) and without CVD (*n* = 241) are shown and compared in Table 2. However, none of the respectively clinical determinants detected differs significantly between the HD patient subgroups.

**Table 2.** Clinical and biochemical characteristics and risk factors of the HD patients with CVD and without CVD.

	HD with CVD	HD without CVD	*p* Value
Number	119	241	–
Gender (M/F)	64/55	117/124	–
Age (years)	67.7 ± 10.3	65.2 ± 14.7	.090
Weight (kg)	59.1 ± 12.5	62.9 ± 47.8	.389
Total protein (g/dL)	6.85 ± 0.54	6.95 ± 0.50	.091
Albumin (g/dL)	4.03 ± 0.32	4.06 ± 0.32	.336
Glucose AC (mg/dL)	128 ± 60	125 ± 58	.648
Total Cholesterol (mg/dL)	163 ± 43	159 ± 35	.365
Triglyceride (mg/dL)	154 ± 91	147 ± 100	.520
AST (U/L)	19.1 ± 8.3	20.0 ± 8.2	.333
ALT (U/L)	17.2 ± 10.1	17.6 ± 10.1	.724
Hct (%)	31.3 ± 3.1	31.9 ± 3.1	.094
MCV (fL)	92.8 ± 7.4	92.4 ± 6.9	.580
Platelet (×10^3^/μL)	193 ± 65	202 ± 61	.174
Hb (g/dL)	10.3 ± 1.1	10.5 ± 1.1	.108
WBC (×10^3^/μL)	7.2 ± 2.2	7.1 ± 2.3	.802
RBC (×10^6^/μL)	3.39 ± 0.42	3.47 ± 0.44	.100
Uric acid (mg/dL)	6.89 ± 1.2	6.98 ± 1.3	.520
BUN (mg/dL)	73.0 ± 17.7	70.2 ± 17.4	.164
Creatinine (mg/dL)	9.02 ± 2.06	9.11 ± 2.12	.683
Na (meq/L)	137 ± 3	137 ± 4	.080
K (meq/L)	4.73 ± 0.58	4.65 ± 0.69	.309
Ca (mg/dL)	7.44 ± 2.47	7.09 ± 2.39	.194
P (mg/dL)	5.46 ± 1.46	5.13 ± 1.47	.049
HD treatment (years)	7.54 ± 6.5	7.80 ± 6.65	.731
Kt/V	1.62 ± 0.24	1.63 ± 0.24	.358

Data were expressed as mean ± SD. Biochemical data were detected before an HD session.

ALT: alanine aminotransferase; AST: aspartate aminotransferase; BUN: blood urea nitrogen; CVD: cardiovascular diseases; Glucose AC: fasting blood glucose; Hb: hemoglobin; Hct: hematocrit; HD: hemodialysis; Kt/V: (*K* – dialyzer clearance of urea; *t* – dialysis time; *V* – volume of distribution of urea, approximately equal to patient’s total body water); MCV: mean corpuscular volume; RBC: red blood count; WBC: white blood count.

### Plasma ACE and ACE2 in the HD patients

Plasma ACE activity in the HD patients was significantly higher than that in the healthy controls (133 ± 40 vs. 101.8 ± 24.8 RFU/μL/h, *p <* .001). Whereas, plasma ACE2 level was significantly lower in the HD patients compared with the healthy controls (13.1 ± 3.6 vs. 8.9 ± 2.6 RFU/μL/h, *p <* .001). Based on the increased ACE and decreased ACE2 activities, a remarkably increased ACE/ACE2 was also obtained in the HD patients compared that in the healthy subjects (15.9 ± 6.2 vs. 8.0 ± 1.6, *p <* .001) (Table 3).

**Table 3.** Levels and comparisons of plasma ACE, ACE2, ACE/ACE2, Ang II and Ang-(1–7) between the controls and HD patients.

	Controls (*n* = 50)	HD patients (*n* = 360)
ACE (RFU/μL/h)	101.8 ± 24.8	133 ± 40***
ACE2 (RFU/μL/h)	13.1 ± 3.6	8.9 ± 2.6***
ACE/ACE2	8.0 ± 1.6	15.9 ± 6.2***
Ang II (ng/mL)	65.9 ± 8.6	105.6 ± 30.6***
Ang-(1–7) (ng/mL)	19.1 ± 3.1	15.0 ± 4.6***

The data were expressed as mean ± SD.

*** *p <* .001, Controls vs. HD patients.

ACE: angiotensin converting enzyme; ACE2: angiotensin converting enzyme; Ang II: angiotensin II; Ang-(1–7): angiotensin 1–7; HD: hemodialysis.

The changes of circulating ACE and ACE2 were observed in the HD patients. We then differentiated these levels in the patients according to different original cause of diseases with and without CVD. Interestingly, significantly higher ACE (145 ± 38 vs. 127 ± 40 RFU/μL/h, *p <* .001) and ACE/ACE2 (21.5 ± 6.3 vs. 13.2 ± 4.0 RFU/μL/h, *p <* .001) and lower ACE2 (7.0 ± 1.5 vs. 9.9 ± 2.5 RFU/μL/h, *p <* .001) in the HD patients with CVD compared to those in the HD patients without CVD were detected (Table 4).

**Table 4.** Levels and comparisons of ACE, ACE2, ACE/ACE2, Ang II and Ang-(1–7) in the HD patients with CVD and without CVD.

	HD with CVD (*n* = 119)	HD without CVD (*n* = 241)
ACE (RFU/μL/h)	145 ± 38	127 ± 40***
ACE2 (RFU/μL/h)	7.0 ± 1.5	9.9 ± 2.5***
ACE/ACE2	21.5 ± 6.3	13.2 ± 4.0***
Ang II (ng/mL)	121 ± 33	98 ± 27***
Ang–(1–7) (ng/mL)	13.8 ± 4.3	15.6 ± 4.7***

Data were expressed as mean ± SD.

*** indicates *p* < .001 compared with the HD with CVD group.

ACE: angiotensin converting enzyme; ACE2: angiotensin converting enzyme; Ang II: angiotensin II; Ang-(1–7): angiotensin 1–7; CVD: cardiovascular diseases; HD: hemodialysis.

### Plasma Ang II and Ang-(1–7) in the HD patients

The patients on HD showed significant increase in plasma concentration of Ang II compared with that in the healthy controls (65.9 ± 8.6 vs. 105.6 ± 30.6 ng/mL, *p* < .001). Whereas, the level of Ang-(1–7) in the HD and control subjects was significantly different and a lower Ang-(1–7) plasma concentration detected in the HD patients (15.0 ± 4.6 vs. 19.1 ± 3.1 ng/mL, *p* < .001) (Table 3).

A higher plasma Ang II and lower Ang-(1–7) level was detected in the HD patients with CVD as compared to the levels in the HD patients without CVD (121 ± 33 vs. 98 ± 27 ng/mL for Ang II, *p* < .001; 13.8 ± 4.3 vs. 15.6 ± 4.7 ng/mL for Ang-(1–7), *p* < .001) (Table 4).

### Correlation coefficients between ACE and ACE2 in the HD patients

In order to evaluate the relationship between the effects of HD patients with and without CVD on ACE and ACE2 levels. We performed multiple linear regression analyses after adjusting for ACE/ACE2, age, body weight and glucose AC. ACE and ACE2 are the significant predictors (*p* < .01) in all of the HD patents, however, ACE/ACE2 ratio is not a significant predictor in the HD patients with CVD compared to it in the HD patients without CVD (Table 5). The result hints that the decreased correlation between ACE and ACE2 in the HD patients may be affected by the complications of CVD.

**Table 5.** Predictors of HD patients with and without CVD by multiple linear regression analysis.

Variable	*β* coefficient	95% CI of *β*	*t*	*p* Value
HD patients with CVD				
ACE	127.087	44.417–209.758	3.049	.003
ACE2	6.655	2.010–11.300	2.842	.005
ACE/ACE2	0.636	−0.521–1.794	1.090	.278
Age	−0.594	−1.360–0.173	−1.536	.128
Body weight	−0.078	−.699–0.543	−0.249	.804
Glucose AC	0.015	−.097–0.127	0.263	.793
HD patients without CVD				
ACE	−120.924	−131.019 to −110.829	−23.600	.000
ACE2	12.862	12.340–13.385	48.532	.000
ACE/ACE2	9.286	8.965–9.607	57.059	.000
Age	0.006	−0.075–0.088	0.155	.877
Body weight	−0.008	−0.033–0.017	−0.658	.511
Glucose AC	−0.014	−0.035–0.006	−1.367	.173

ACE: angiotensin converting enzyme; ACE2: angiotensin converting enzyme; Ang II: angiotensin II; Ang-(1–7): angiotensin 1–7; CVD: cardiovascular diseases; Glucose AC: fasting blood glucose; HD: hemodialysis.

**Figure 1.**
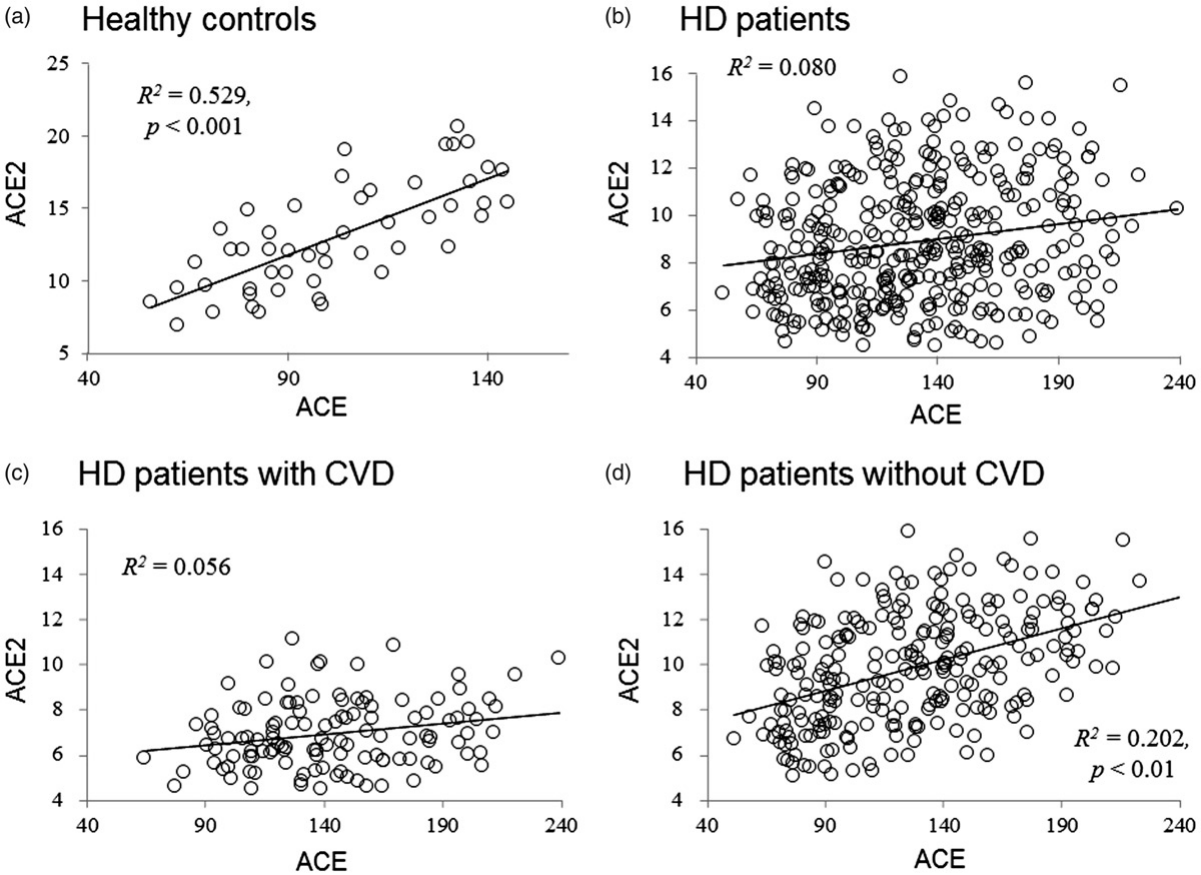
The relation between plasma ACE and ACE2 activity in the healthy controls (a), all of HD patients (b), the HD patients with CVD (c) and the HD patients without CVD (d). Healthy controls (*n* = 50), HD patients (*n* = 360), HD patients with CVD (*n* = 119), HD patients without CVD (*n* = 241). ACE: angiotensin converting enzyme; ACE2: angiotensin converting enzyme; CVD: cardiovascular diseases; HD: hemodialysis.

In the healthy controls, a pretty high positive correlation between plasma ACE and ACE2 activity was observed (*R*^2^ = 0.529, *p <* .001). However, there was a relatively lower correlation coefficient obtained between plasma ACE and ACE2 activity in the HD patients (*R*^2^ = 0.080) (Figure 1). The decreased correlation coefficient between ACE and ACE2 because increased ACE and decreased ACE2 activities in the HD patients. It is noted that the decreased correlation between ACE and ACE2 was major contributed from the HD patients with CVD compared to that in the HD patients without CVD (*R*^2^ = 0.056 vs. *R*^2^ = 0.202) (Figure 1).

### Changes during HD session

The parameters of BUN, creatinine, ACE, ACE2, ACE/ACE2, Ang II and Ang-(1–7) levels in the HD patients before (pre-DH) and after (post-HD) a single HD session were determined and compared. As expectation, the results show the significantly reductions of BUN and creatinine in the post-HD compared those in the pre-HD (Table 6).

**Table 6.** The changes of ACE, ACE2, ACE/ACE2, Ang II and Ang-(1–7) in the HD patients (*n* = 360) before (pre-HD) and after (post-HD) a single HD session.

	Pre-HD	Post-HD
BUN (mg/dL)	71.1 ± 17.5	17.3 ± 5.6***
Creatinine (mg/dL)	9.08 ± 2.10	2.96 ± 1.10***
ACE (RFU/μL/h)	133 ± 40	140 ± 45*
ACE2 (RFU/μL/h)	8.9 ± 2.6	9.0 ± 2.8
ACE/ACE2	15.9 ± 6.2	16.9 ± 7.6
Ang II (ng/mL)	106 ± 31	111 ± 31*
Ang-(1–7) (ng/mL)	15.0 ± 4.6	14.9 ± 4.1

Data were expressed as mean ± SD.

* *p* < .05 and

*** *p* < .001, pre-HD vs. post-HD.

ACE: angiotensin converting enzyme; ACE2: angiotensin converting enzyme; Ang II: angiotensin II; Ang-(1–7): angiotensin 1–7; HD: hemodialysis; pre-HD: before hemodialysis; post-HD: after hemodialysis.

Plasma ACE was increased (pre-HD 133 ± 40 RFU/μL/h vs. post-HD 140 ± 45 RFU/μL/h, *p <* .05), but ACE2 was similar (pre-HD 8.9 ± 2.6 RFU/μL/h vs. post-HD 9.0 ± 2.8 RFU/μL/h, *p =* .576) in the HD patients after a single HD session. A trend of increased ACE/ACE2 ratio (pre-HD 15.9 ± 6.2 vs. post-HD 16.9 ± 7.6, *p <* .05) could be calculated (Table 6). Similar results were also observed in the changes of Ang II and Ang-(1–7), an increased Ang II (pre-HD 106 ± 31 vs. post-HD 111 ± 31 ng/mL, *p <* .05) and similar Ang-(1–7) (pre-HD 15.0 ± 4.6 vs. post-HD 14.9 ± 4.1 ng/mL, *p =* .68) was detected in the HD patients after a single HD session (Table 6).

### Changes during HD session in the patients with and without CVD

We then differentiated these levels in the HD patients with and without CVD. In the HD patients with CVD, plasma ACE activity was significantly increased from pre-HD: 145 ± 38 RFU/μL/h to post-HD 160 ± 43 RFU/μL/h (*p* < .01) and plasma ACE2 activity was decreased (pre-HD: 7.0 ± 1.5 RFU/μL/h vs. pre-HD: 6.5 ± 1.4 RFU/μL/h, *p* < .05) due to HD treatment. In the HD patients without CVD, both of plasma ACE and ACE2 levels were not significantly different between the pre-HD and post-HD detections (Table 7). In Table 7, the data show that circulating ACE/ACE2 ratio in the HD patient groups with CVD was significantly increased after a single HD session (*p* < .001), but the ratio of ACE/ACE2 in the HD patients without CVD were similar before and after a single HD session (*p* = .610).

**Table 7.** The changes of ACE, ACE2, ACE/ACE2, Ang II and Ang-(1–7) in the HD patients with CVD (*n* = 119) and without CVD (*n* = 241) before (pre-HD) and after (post-HD) a single HD session.

	With CVD pre–HD	With CVD post–HD	Without CVD pre–HD	Without CVD post–HD
ACE (RFU/μL/h)	145 ± 38	160 ± 43**	127 ± 40	130 ± 43
ACE2 (RFU/μL/h)	7.0 ± 1.5	6.5 ± 1.4*	9.9 ± 2.5	10.3 ± 2.4
ACE/ACE2	21.5 ± 6.3	25.0 ± 6.5***	13.2 ± 4.0	12.9 ± 4.1
Ang II (ng/mL)	121 ± 33	132 ± 27**	98 ± 27	100 ± 27
Ang –(1–7) (ng/mL)	13.8 ± 4.3	12.9 ± 2.6	15.6 ± 4.7	15.8 ± 4.3

Data were expressed as mean ± SD.

* *p* < .05.

** *p* < .01 and

*** *p* < .001, pre-HD vs. post-HD in the same group, the HD patients with CVD and without CVD.

ACE: angiotensin converting enzyme; ACE2: angiotensin converting enzyme; Ang II: angiotensin II; Ang-(1–7): angiotensin 1–7; CVD: cardiovascular diseases; HD: hemodialysis; pre-HD: before hemodialysis; post-HD: after hemodialysis.

In the HD patients with CVD, Ang II level was significantly increased from its pre-HD 121 ± 33 ng/mL to post-HD 132 ± 27 ng/mL (*p* < .01). However, pre-HD Ang-(1–7) and post-HD Ang-(1–7) levels were similar in this group. In the HD patients without CVD, both Ang II and Ang-(1–7) levels were insignificantly changes from its pre-HD levels to its post-HD levels (Table 7).

## Discussion

We reported increased ACE activity and decreased ACE2 activity in the circulation of uremic patients on HD (i.e., HD patients). The result indicated that appropriate balance between ACE and ACE2 activity is demolished, i.e., imbalanced ACE/ACE2. Among of the HD patients with different original causes, a markedly imbalanced ACE/ACE2 was observed in the patients with CVD. In the course of an HD session, the circulating levels of increased ACE and decreased ACE2 in the HD patients with CVD, but not in the patients without CVD, were measured. These results are to supposedly lead in the elevation of circulating ACE/Ang II/ATR1 axis and reduction of ACE2/Ang-(1–7)/Mas axis after a single HD session in the HD patients with CVD. In the 1980’s, several studies evaluated the circulating ACE levels in HD patients. Malik et al. [20] wrote a detail review on this topic. In 1979, Patel et al. [21] performed one of the first known evaluations of the ACE level in HD patients. They pointed that serum ACE activity was increased in 58% of 19 patients with CKD on long-term HD. Elevated circulating ACE in HD patients were following reported by several research teams [22–24]. However, insignificant difference on circulating ACE between HD patients and control subjects has been also reported [25,26]. It is needed to consider limited HD patients enrolled in the above studies. Recently, a study with a large population including 546 patients on HD and 568 controls were performed by Anguiano et al. [19]. The study indicated that a significantly elevated ACE activity in HD patients compared that in control group. In line with our findings, it could be concluded that most studies have showed that higher circulating ACE in the HD patients on regular HD treatment. Moreover, we have provided the advanced evidence of a higher Ang II level in these HD patients in this study (Table 3).

There were different mechanisms to explain why circulating ACE activity is increased in HD patients. Based on the finding in our study, the plasma ACE activity in the HD patients with CVD was significant higher than that in the HD patients without CVD (Table 4), we supported that elevated ACE in HD patients is possibly because of vascular endothelial damage [22]. In spite of this proposition is still incomplete, but our increasing understanding of the complicated nature of RAS and its effects in CKD, especially in the CKD patient with history CVD.

In this study, we revealed a statistically significant increase in the plasma ACE level in the HD patients after an HD session (Table 6). The result is consistent with the most previous works [26–28]. It is important to know whether the elevation of ACE in HD patients is due to CKD itself, HD session and/or other diseases, such as CVD. However, the mechanisms underlying the ACE regulation are complex and related to multiple interactions with disease progression of the CKD and CVD in HD patients [20]. Nielsen et al. [27] also suggested that increased serum ACE was due to a complement-mediated sequestration of leukocytes within the pulmonary vasculature and the leukocytes accumulation could lead to injury of the vascular endothelium with interstitial edema. The effect of different dialyzer membranes on serum ACE in HD patients has been studied and concluded that circulating ACE change in the HD patients is less a cause of the HD procedure itself [28].

Compared to the studies on ACE, only a small number of studies have directly examined the circulating ACE2 activity in HD patients. Soler et al. [29] reported that serum ACE2 activity was detectable in kidney transplantation (Kt) recipients and was increased in Kt recipients with ischemic heart disease as compared to the Kt recipients without ischemic heart disease [29]. Roberts et al. [30] showed that among patients with CKD, plasma ACE2 activity is lower in those undergoing HD for ESRD patients when compared with pre-dialysis patients with CKD or Kt patients. Note that in above two studies, healthy controls did not be enrolled in the studies for the ACE2 determination and comparison. Recently, Úri et al. [31] displayed that circulating ACE2 activities were increased in patients with heart failure and hypertension, while ACE2 reduced with the amelioration of the heart failure after Cardiac resynchronization therapy (CRT) [31]. The activity of ACE2 was reduced during acute ischemic stroke, and improving elevates by 3 d post-stroke [32]. Previous study demonstrated that the serum ACE2 level can significantly predict the magnitude of coronary artery calcification (CAC), and indicated that elevated ACE2 may be involved in vascular calcification in patients on maintenance hemodialysis therapy [33]. Anguiano et al. [19] reported a study with larger sample size (*n* = 2572) of subjects. In the study, the subjects, including control, CKD patients at stages 3–5 (CKD3–5) and CKD patients in dialysis (CKD5D), were grouped and all of the subjects had no history of CVD. They found that plasma ACE2 activity in the CKD5D patients was significantly decreased compared those in the CKD3–5 patients and control population [19]. This result was in agreement with previous result [30] and also similar to our results shown in this study (Table 4). We agree the comment from Wysocki et al. [34] who proposed that reduced plasma ACE2 activity in HD patients may be another piece in the conundrum of factors involved in hypertension and cardiovascular morbidity for the HD patients.

Logically, measurements of circulating ACE and ACE2 in the HD patients should show no differences between their pre-HD and post-DH levels, because that the enzymes are not removed by dialysis due to its large molecular size. As mentioned above, the increased ACE and decreased ACE2 plasma levels in the HD patients with CVD after a single HD session was detected in our study. However, such changes on plasma ACE and ACE2 levels in the HD patients have no significant change from pre-HD to post-HD during HD session (Table 7). According to our best knowledge, we are first to report the changes of circulating ACE and ACE2 activity in the HD patients during a single HD session. It cannot be excluded that any endogenous inhibitors or enhancers with a small molecular weight that can be remove during HD treatment. For example, the presence of a small molecular endogenous inhibitor of ACE2 in human plasma has been reported [35]. HD itself could potentially alter the levels of the ACE2 inhibitor in plasma owing to its small molecular size. Removal of the inhibitor during a single HD procedure could increase plasma ACE2 activity. However, this proposition is still to be tested. If the plasma ACE2 inhibitor indeed does not play a role in the patients during HD session, the question that remains is what causes the observed changes in plasma ACE2 activity in the HD patients with or without CVD. The significance of plasma ACE2 activity, however, is not totally clear, since ACE2 is mainly a tissue enzyme and its levels in the circulation, unlike the levels of ACE, are relatively low [34]. ACE2 produced and shed into the circulation from kidneys is a possibility since ACE2 activity is very high in the kidney [29,34]. In fact, in pathological states in humans, such as CVD and diabetes accompanied by vascular complications, circulating ACE2 activity is still augmented [36,37].

Various studies have used animal models of kidney injury to investigate the renal pathological changes of ACE/ACE2 ratio [35]. However, clinical studies in human are very limited. Kidney biopsies from patients have indicated that hypertensive patients have higher ACE/ACE2 mRNA ratios [11]. In a hypertensive model, increased ACE and decreased ACE2 along with a higher ACE/ACE2 ratio in hypertensive kidneys appeared to favor Ang II generation, leading to hypertensive renal damage [38]. High ACE/ACE2 ratios in patients with type 2 diabetes and overt nephropathy were detected; thus, such changes might play a role in renal damage [39]. In a study of patients with hypertensive nephrosclerosis, Wang et al. [40] detected a correlation between the glomerular ACE/ACE2 protein ratio and the extent of glomerulosclerosis and an inverse correlation between the glomerular ACE/ACE2 protein ratio and the estimated glomerular filtration rate (eGFR). Conversely, no associations were detected between the tubulointerstitial ACE/ACE2 ratio and histological or clinical parameters [41]. Pohl et al. [41] demonstrated that whilst the ACE2 is expressed along the entire renal tubular segment ACE is only expressed in the brush-border membrane of the late proximal tubules and they suggested that surface expression of ACE and ACE2 differed as a function of endocytosis. Batlle et al. [13] suggested that as ACE and ACE2 are regulated via different mechanisms and the ACE/ACE2 ratio could be misleading. Together, the findings of these studies indicate that increases in the ACE/ACE2 ratio induced via the ACE/Ang II/AT1 axis have a significant influence on the development of severe kidney damage. On the other hand, renal ACE/ACE2 ratio data should be interpreted carefully, as ACE and ACE2 are regulated via independent mechanisms [42–44]. As we mentioned above, only a small number of studies have examined the activity of circulating ACE2 in humans with related CKD. Therefore, the regulatory mechanisms focused on the alternating ACE and ACE2 regulation in the HD patients with or without CVD remains to be further explored.

## Conclusions

Increased ACE and decreased ACE2 to lead imbalanced ACE/ACE2 ratio was detected in the circulation of HD patients, particularly those with history CVD. The alterations of circulating ACE and ACE2 activity in the HD patients might be related with impaired renal and cardiovascular functions, or HD session. In the course of a single HD session, the circulating levels of increased ACE and decreased ACE2 in the HD patients with CVD, but not in the patients without CVD, were observed. This may provide the basis for the assessment of altered RAS axis, including ACE/Ang II/ATR1 and ACE2/Ang-(1–7)/Mas, related with the pathophysiological state during HD session. To our knowledge, this is one of the first studies to demonstrate the parallel alterations of ACE and ACE2 activities in HD patients and the patients with CVD. Although our preliminary observations merit further research, these results provide the importance of hypertension control for the HD patients with history CVD during HD session.

## Ethical approval

The study protocol (IRB20130811R) was reviewed and approved by the Bioethics Committee of Shin Kong Wu Ho-Su Memorial Hospital (Taiwan).

## Informed consent

Informed consent was obtained from parents/guardians of all individual participants included in the study.

## Supplementary Material

Supplementary Table
