# Supplementary material for: Imbalanced plasma ACE and ACE2 level in the uremic patients with cardiovascular diseases and its change during a single hemodialysis session
Source: Ren Fail. 2017 Nov 20;39(1):719–28. doi: 10.1080/0886022X.2017.1398665 (PMC6446170; doi:10.1080/0886022X.2017.1398665)
Supplement: Supplementary Table [file IRNF_A_1398665_SM2794.pdf]

Supplementary information

Imbalanced plasma ACE and ACE2 level in the uremic patients with cardiovascular diseases and its change during a single hemodialysis session

Chung-Wei Yang [a,b], Li-Che Lu [c†], Chia-Chu Chang [d,e], Ching-Chang Cho[a], Wen-Yeh Hsieh [f,g], Chin-Hung Tsai [a], Yi-Chang Lin [a], Chih-Sheng Lin [a,*]

[a] Department of Biological Science and Technology, National Chiao Tung University, Hsinchu, Taiwan

[b] Division of Nephrology, Department of Internal Medicine, National Taiwan University Hospital Hsinchu Branch, Hsinchu, Taiwan

[c] Division of Nephrology, Department of Internal Medicine, Shin Kong Wu Ho-Su Memorial Hospital, Taipei, Taiwan

[d] Division of Nephrology, Department of Internal Medicine, Changhua Christian Hospital, Changhua, Taiwan

[e] School of Medicine, Chung-Shan Medical University, Taichung, Taiwan

[f] Division of Chest Medicine, Department of Internal Medicine, Hsinchu Mackay Memorial Hospital, Hsinchu, Taiwan

[g] Department of Senior Citizen Service Management, Minghsin University of Science and Technology, Hsinchu, Taiwan

[†] These authors contributed equally to the manuscript

***** Corresponding author (lincs@mail.nctu.edu.tw)

Table S1. Comparisons among the HD with CVD, HD without CVD and healthy controls.

**Group**	**Gender (M/F)**	**Age**	**Inclusion criteria**	**Exclusion criteria**
Healthy controls	27/23	63.6 ± 13.9	1. Healthy subjects<br>2. Non-smoking volunteers<br>3. Age and gender that matched for HD patients	1. Age < 20 years<br>2. Any symptoms and signs indicative of either renal or cardiovascular diseases
HD with CVD	64/55	67.7 ± 10.3	1. HD patients at CKD Stage 5<br>2. Hemodialysis > 0.25 years<br>3. Clinical symptoms of cardiovascular diseases	1. Age < 20 years<br>2. ACEI uses<br>3. Positive serum marker of HIV<br>4. Alcohol or illicit drug abusers
HD without CVD	117/124	65.2 ± 14.7	1. HD patients at CKD Stage 5<br>2. Hemodialysis > 0.25 years	1. Age < 20 years<br>2. ACEI uses<br>3. Positive serum marker of HIV<br>4. Alcohol or illicit drug abusers<br>5. Clinical symptoms of cardiovascular diseases

HD: hemodialysis, CVD: cardiovascular diseases, CKD: chronic kidney diseases,
ACEI: angiotensin converting enzyme inhibitor, HIV: human immunodeficiency virus
